# Performance evaluation of a commercial multiplex pathogen panel for the diagnosis of pediatric joint infections

**DOI:** 10.1128/jcm.00278-25

**Published:** 2025-06-02

**Authors:** Lawson Copley, Gina Lee, Mary Villani, Naureen Tareen, Laura M. Filkins

**Affiliations:** 1 Department of Pediatric Orthopaedic Surgery, Children’s Medical Center of Dallas and University of Texas Southwestern Medical Center 12334, Dallas, Texas, USA; 2 Department of Pediatrics, Division of Infectious Diseases, University of Texas Southwestern Medical Center 196285, Dallas, Texas, USA; 3 Department of Pediatric Orthopaedic Surgery, Children’s Health System of Texas, Dallas, Texas, USA; 4 Department of Pathology, University of Texas Southwestern Medical Center 12334, Dallas, Texas, USA; Cleveland Clinic, Cleveland, Ohio, USA

**Keywords:** joint infection, pediatric infectious disease, multiplex panel

## Abstract

This study evaluates the performance of the BIOFIRE Joint Infection (JI) Panel compared to joint fluid culture and/or 16S rRNA PCR, followed by Sanger sequencing (16S PCR/S) for the diagnosis of joint infections in pediatric patients. An on-panel organism was detected by standard-of-care joint (SOCj) studies (joint fluid culture with or without 16S PCR/S, as ordered by the treating physician) in 29/65 samples (44.6%), and the same organism was detected by the BIOFIRE JI panel in all samples. The cumulative positive percent agreement in the detection of on-panel genus or species-level targets by the BIOFIRE JI panel was 100% (36/36), 100% (25/25), and 100% (27/27), and the negative percent agreement was 99.7% (1,974/1,979), 99.2% (1,974/1,990), and 99.7% (1,303/1,307) compared to detection by SOCj, joint fluid culture only, or 16S PCR/S only, respectively. The potential clinical impact of employing the BIOFIRE JI panel was predicted by retrospective adjudication using a study-specific rubric. We predicted that 27.7% (18/65) of BIOFIRE JI panel results could have had a positive impact on patient care. One case (1.5%) was predicted to potentially have a negative impact, and three cases (4.6%) were adjudicated to have an unknown impact. All organisms detected by 16S PCR/S, but not by culture, were also detected by the BIOFIRE JI panel during this study.

**IMPORTANCE:**

The BIOFIRE JI Panel has been limitedly evaluated in pediatric patients. Our study shows a strong agreement between the BIOFIRE JI panel and culture and/or 16S rRNA PCR with Sanger sequencing for the detection of the most common pathogenic causes of joint infection in children. The faster time to results of the BIOFIRE JI panel has the potential to guide optimal treatment faster than conventional methods.

## INTRODUCTION

Joint infections in children include primarily septic arthritis with or without osteomyelitis. Infrequently, postoperative or prosthetic joint infections can occur. In many cases, no causative organism is detected, in part due to low sensitivity of culture (21%–70% positivity rates) (1, 2). Timely and accurate identification of the causative organism is required to optimize treatment, minimize potential joint damage, mitigate poor clinical outcome, and avoid unnecessary exposure to broad-spectrum antibiotics.

Bacterial pathogens can be challenging to recover in culture due to several causes including the following: low bacterial load, bactericidal properties of synovial fluid, phagocytosis and killing by the immune response, pretreatment with antibiotics, and the fastidious nature of some organisms (e.g., *Kingella kingae*) (3, 4). Nucleic acid amplification-based methods, such as targeted PCR or 16S rRNA PCR followed by sequencing in addition to culture, can increase the detection and identification of infecting organisms compared to culture alone (5–7). At our institution, it was previously shown that 16S rRNA PCR with Sanger sequencing (16S PCR/S) enabled bacterial identification in 20.6% of culture-negative cases (8).

In 2022, bioMérieux received *de novo* FDA authorization from the United States FDA for its BIOFIRE Joint Infection (JI) panel: a broad multiplex panel simultaneously targeting 29 bacterial pathogens, two fungal pathogens, and eight antimicrobial resistance genes for testing synovial fluid from patients with suspected joint infection. Reported clinical performance of pathogen detection by the BIOFIRE JI panel ranges from 71.4% to 100% sensitivity or positive percent agreement and 94.8% to 100% specificity or negative percent agreement for on-panel targets compared to conventional culture-based testing (9–12). The clinical sensitivity of the BIOFIRE JI panel was shown to be lower than that of conventional microbiology testing (41.4% compared to conventional testing) and lower than that of 16S PCR followed by next-generation sequencing (56% BIOFIRE JI panel versus 93% by sequencing) for the diagnosis of prosthetic joint infections in adults (13, 14). The lower sensitivity of the BIOFIRE JI panel has been attributed to off-panel causes of infection, especially *Staphylococcus epidermidis*.

The performance of the BIOFIRE JI panel in the pediatric population is not well-reported. In this study, we evaluated the analytic performance and the potential clinical impacts of the BIOFIRE JI panel compared to conventional culture and/or 16S PCR/S for the diagnosis of joint infections at our pediatric institution.

## MATERIALS AND METHODS

### Joint fluid specimens and enrollment

Patients under evaluation for joint infection at Children’s Health System of Texas were considered for inclusion in the study between August 1, 2020 and October 1, 2023. Synovial joint fluid specimens were collected during standard-of-care procedures. Specimens were collected in a sterile syringe without additives. Extra or residual fluid with a minimum volume of 200 µL was stored and refrigerated for up to 7 days and then was directly tested by the BIOFIRE JI panel or was aliquoted to DNAase-free, additive-free vials and frozen at −80°C prior to testing.

Patients were either identified and consented prior to a joint aspiration procedure by the orthopedic physician or were enrolled via waiver of consent if residual synovial joint fluid was available after standard-of-care testing was processed, and a joint fluid culture was ordered. This study was reviewed and approved by the University of Texas Southwestern Medical Center Institutional Review Board.

### Clinical diagnostic testing

Standard-of-care microbiologic joint infection (SOCj) testing was defined as any microbiologic testing performed on joint fluid obtained from enrolled patients. At our institution, aerobic body fluid culture with or without 16S PCR/S (University of Washington Molecular Diagnostics Laboratory) is routinely performed. For aerobic body fluid culture, 50–100 µL of the joint fluid specimen was inoculated to each a chocolate agar, blood agar, and MacConkey agar plate, and 0.5–3 mL was inoculated to a BD BACTEC Peds Plus blood culture bottle. Plates and bottles were incubated for 5 days before reporting “no growth” in culture. Anaerobic bacterial culture, fungal culture, and acid-fast bacillus culture were also evaluated as part of SOCj testing if ordered on the corresponding joint fluid specimen. However, these were infrequently ordered, and none were positive in this study. BIOFIRE JI panel results were compared to SOCj only if the joint fluid specimen was from the same joint and collected during the same procedure. The complete standard-of-care (cSOC) testing included all microbiologic testing procedures that contributed to treatment decisions (e.g., blood cultures, bone or joint tissue, and abscess cultures).

### BIOFIRE JI panel testing

All BIOFIRE JI panel testing procedures were performed using the FDA-approved BIOFIRE JI panel formulation following the manufacturer’s instructions. Synovial fluid (200 µL) was tested from each enrolled specimen. Specimens enrolled prior to FDA approval of the BIOFIRE JI panel were frozen and stored until testing. For frozen specimen testing, samples experienced a maximum of one thawing cycle before BIOFIRE JI panel testing. BIOFIRE JI panel results were not provided to the clinicians or used for patient care.

### Potential clinical impact analysis

The potential clinical impact of the BIOFIRE JI panel results was assessed for each specimen tested using study-specific definitions and criteria. Predictions were made based on the chart review of the cSOC results, timing of results, type of therapy and therapy rationale, and timing of therapy decisions. The therapy rationale included joint and non-joint infection clinical concerns that were likely to drive treatment with or without BIOFIRE JI panel results (e.g., broad empiric therapy in a complex patient with concern for sepsis). The type of impacts (positive, negative, no, or unknown) and degree of positive impact (low, moderate, high, and critical) were assigned following the predicted impact matrix in Table 1. To determine the potential differences in time to results between cSOC and the BIOFIRE JI panel, we assumed BIOFIRE JI panel results would be reported in the patient chart 3 h after specimen collection. “Likely to optimize” criteria included the following: i) if cSOC organism identification drove management recommendations before antimicrobial susceptibility testing results were reported, we assumed an organism identification by the BIOFIRE JI panel would similarly be used or ii) if SOCj studies were negative, but bone or tissue samples from the same procedure were positive and drove management, we assumed that positive BIOFIRE JI panel results would similarly drive management or iii) if empiric therapy was narrowed at discharge prior to obtaining a pathogen identity, we assumed therapy would have been targeted as per an earlier identification by the BIOFIRE JI panel in a stable, otherwise well patient. “Not likely to optimize” criteria included the following: i) if non-musculoskeletal studies drove management (such as in complex patients being treated for other concerns or patients with preceding bacteremia), and/or ii) if antibiotics were not optimized until phenotypic antimicrobial susceptibility testing results were available, and/or iii) empiric therapy was the same as targeted therapy, it was assumed that the BIOFIRE JI panel results would not have altered the management. When assessing the potential clinical impact, we set the standard of care as the reference and assumed that the clinical interpretation of the cSOC testing by the patient’s care team was correct. Each case was adjudicated by two study personnel [LF (clinical microbiologist) and GL or MV (infectious disease providers)], and discrepant adjudications were resolved by a third adjudicator (MV or GL).

**TABLE 1:** Potential impact clinical adjudication matrix^*a*^

Impact	Criteria
No Impact	Empiric therapy was the same as targeted therapy OR Negative JI panel but clinical evidence of infection and empiric therapy given despite negative cSOC results OR Other microbiologic workup would guide optimization before/instead of JI panel results OR Other clinical concerns guided broader treatment selection OR Other clinical and/or laboratory results were required before making a clinical diagnosis of infectious versus non-infectious
Low Positive	Empiric therapy was adequate AND Organism would be identified < 48 h sooner AND Likely to optimize antibiotics
	Empiric therapy was not adequate AND Organism would be identified < 12 h sooner AND Likely to optimize antibiotics
Moderate Positive	Empiric therapy was adequate AND Organism would be identified ≥ 48 h sooner AND Likely to optimize antibiotics
	Empiric therapy was not adequate AND Organism would be identified ≥ 12 h sooner AND Patient not critically ill/clinically resolved despite suboptimal therapy
High Positive	Empiric therapy was not adequate AND Organism would be identified ≥ 12 h sooner AND Critically ill patient with prolonged or complicated recovery path
	Organism would be identified ≥ 24 h sooner AND Opportunity to discharge ≥ 1 d sooner (i.e., admission specifically for intravenous antimicrobials)
	Additional invasive procedures (e.g., requiring general anesthesia or operating room procedures) could have been avoided due to JI panel results (all adjudicators must agree)
Critical Positive	Clinically adjudicated to be potentially “lifesaving” (all adjudicators must agree)
Negative	JI panel results were different from cSOC and may have guided clinical management in a potentially harmful or suboptimal direction
Unknown	Organism detected by JI panel was different than cSOC results AND Organism detected by JI panel was adjudicated to be a plausible cause of the patient's clinical syndrome AND Empiric therapy was different than targeted therapy

^*a*^ cSOC, complete standard-of-care microbiology testing (included all microbiologic testing that contributed to treatment decisions); JI panel, BIOFIRE Joint Infection panel.

### Statistical analysis

Positive and negative percent agreement were calculated comparing target-level or sample-level detection by the BIOFIRE JI panel to SOCj testing, joint fluid culture only, and 16S PCR/S only or cSOC. The odds ratio (OR) and statistical significance of positive organism detection were compared between samples from patients administered antibiotics within 24 h before the joint fluid collection versus no antibiotics using Fisher’s exact test (GraphPad Prism 10.3.0).

## RESULTS

### Patient and sample characteristics

Sixty-five synovial joint fluid specimens were evaluated from 63 patients, collected during 64 different infection evaluations (Table 2). From one patient, two joint fluid specimens were tested that were collected during two different procedures separated by a duration of 1.5 years. From a second patient, two joint fluid specimens were tested that were collected from two different joints during the same operating room procedure. Almost all specimens were collected from native joints (64/65, 98.5%).

**TABLE 2:** Characteristics of enrolled patients and tested samples^*a*^

	Total count
Samples	65
Patients	63
Operating room procedures	64

	Number of patients (%)
Sex of patient (*n* = 63)	
Female	28 (44.4)
Male	35 (55.6)
Age at time of the procedure (*n* = 64)	
<0.5 y	4 (6.3)
0.5–4 y	24 (37.5)
5–12 y	15 (23.4)
13–18 y	20 (31.3)
>18 y	1 (1.6)
Average age [range]	7.6 y [0.13–19 y]

	Number of samples (%)
Joint fluid sources (*n* = 65)	
Left ankle	0 (0)
Left elbow	2 (3.1)
Left hip	4 (6.2)
Left knee	25 (38.5)
Right ankle	4 (6.2)
Right elbow	2 (3.1)
Right hip	4 (6.2)
Right knee	24 (36.9)
Type of joint (*n* = 65)	
Native	64 (98.5)
Prosthetic	1 (1.5)
Pretreatment with antibiotics (*n* = 65)	
Antibiotics within 24 h prior to aspiration	24 (36.9)
No antibiotics within 24 h prior to aspiration	41 (63.1)
Organism detection by SOCj (*n* = 65)	
Only an on-panel organism detected by SOCj	26 (40.0)
Only an off-panel organism detected by SOCj	3 (4.6)
Both on- and off-panel organisms detected by SOCj	3 (4.6)
No organism detected by SOCj	33 (50.8)
SOCj testing composition (*n* = 65)	
Joint fluid culture and 16S PCR/S	46 (70.8%)
Joint fluid culture only	19 (29.2%)

^*a*^ SOCj, standard-of-care joint fluid microbiologic studies (including joint fluid culture with or without 16S PCR/S, as ordered by the clinical team); 16S PCR/S, 16S rRNA PCR followed by Sanger sequencing.

The age of patients at the time of the joint aspiration procedure ranged from 1 month to 19 years, with an average age of 7.6 years. Most patients were male (55.6%, 35/63). For 63.1% (41/65) of the tested synovial fluid specimens, no antibiotics were administered to the patient within 24 hours before the joint aspiration procedure. Knee joints were the most common sources tested in this study (75.4%, 49/65 specimens).

### SOCj test results

As defined in the *Methods*, SOCj testing included the combined results from joint fluid culture with or without joint fluid 16S PCR/S. Aerobic joint fluid culture was always performed. Both joint fluid culture and 16S PCR/S were performed on 70.8% of samples (46/65) (Table 2).

One or more organisms were detected by SOCj in 49.2% of samples (32/65) (Table 2). An organism that is also targeted by the BIOFIRE JI panel (i.e., an “on-panel” organism) was detected by SOCj in 44.6% of samples (29/65). Only organisms that are not targeted by the BIOFIRE JI panel (i.e., “off-panel” organisms) were detected in three samples (4.6%, 3/65). In three samples (4.6%, 3/65), both an on-panel and off-panel organism were detected. The results of all samples with an off-panel organism detected are described in Table S1. No organism was detected by SOCj in 50.8% of samples (33/65).

16S PCR/S results were positive in 47.8% (22/46) of the samples tested; an on-panel organism was detected in 21/22 of those samples. In 32.6% (15/46) of the tested samples, the same organism was detected by culture and 16S PCR/S. Seven organisms were detected by 16S PCR/S, which were not detected in the joint fluid culture and were all interpreted to be clinically significant by the treating team, including the following: three *K. kingae*, two *Streptococcus pyogenes*, one *Streptococcus pneumoniae*, and one *Streptococcus agalactiae.*

### Target-level analytic performance evaluation of the BIOFIRE JI panel

The percent agreement of BIOFIRE JI panel results were compared to those of SOCj (i.e., the combined results from joint fluid culture with or without joint fluid 16S PCR/S), joint fluid culture only, and 16S PCR/S only results (Table 3). The cumulative organism target-level positive percent agreement in the detection of on-panel organism targets by the BIOFIRE JI panel was 100% (36/36), 100% (25/25), and 100% (27/27) compared to detection by SOCj, joint fluid culture only, or 16S PCR/S only, respectively. This corresponded with the results of 29 samples evaluated with the SOCj, 22 samples for joint fluid culture only, and 21 samples for 16S PCR/S only comparators that were positive with an on-panel organism. In seven SOCj-positive samples, both a species-level and corresponding genus-level target were detected by the BIOFIRE JI Panel, including six samples with *Streptococcus* species and one sample with *Candida* species. Notably, the BIOFIRE JI panel detected all organisms that were detected by 16S PCR/S, but that were not recovered in culture (three *K. kingae*, two *S. pyogenes*, one *S. pneumoniae*, and one *S. agalactiae*; Table 3). Six off-panel organisms were detected by SOCj (four samples with *S. epidermidis*, one *Staphylococcus capitis*, and one *Bacillus* species).

**TABLE 3:** Organism detection performance by the BIOFIRE Joint Infection Panel compared to standard of care methods^*a*^^,^^*b*^

	Positive percent agreement of BIOFIRE Joint Infection Panel to comparator methods			Negative percent agreement of BIOFIRE Joint Infection Panel to comparator methods		
	SOCj testing	Joint fluid culture only	16S PCR/S only	SOCj testing	Joint fluid culture only	16S PCR/S only
On-panel targets						
*Staphylococcus aureus*	100% (14/14)	100% (14/14)	100% (9/9)	100% (51/51)	100% (51/51)	97.3% (36/37)
MRSA	75% (3/4)	75% (3/4)	Not targeted	100% (10/10)	100% (10/10)	Not targeted
*Kingella kingae*	100% (3/3)	NA (0/0)	100% (3/3)	96.8% (60/62)	92.3% (60/65)	95.3% (41/43)
*Streptococcus* species	100% (6/6)	100% (2/2)	100% (6/6)	98.3% (58/59)	92.1% (58/63)	100% (40/40)
*Streptococcus pyogenes*	100% (4/4)	100% (2/2)	100% (4/4)	100% (61/61)	96.8% (61/63)	100% (42/42)
*Streptococcus pneumoniae*	100% (1/1)	NA (0/0)	100% (1/1)	98.4% (63/64)	96.9% (63/65)	100% (45/45)
*Streptococcus agalactiae*	100% (1/1)	NA (0/0)	100% (1/1)	100% (64/64)	98.5% (64/65)	100% (45/45)
*Salmonella* species	100% (1/1)	100% (1/1)	100% (1/1)	98.4% (63/64)	98.4% (63/64)	100% (45/45)
*Escherichia coli*	100% (2/2)	100% (2/2)	100% (2/2)	100% (63/63)	100% (63/63)	100% (44/44)
*Staphylococcus lugdunensis*	100% (1/1)	100% (1/1)	NA (0/0)	100% (64/64)	100% (64/64)	100% (46/46)
*Neisseria gonorrhoeae*	100% (1/1)	100% (1/1)	NA (0/0)	100% (64/64)	100% (64/64)	97.8% (45/46)
*Candida* species	100% (1/1)	100% (1/1)	Not targeted	100% (64/64)	100% (64/64)	Not targeted
*Candida albicans*	100% (1/1)	100% (1/1)	Not targeted	100% (64/64)	100% (64/64)	Not targeted
All other organism targets (19 targets)	NA (0/0)	NA (0/0)	NA (0/0)	100% (1,235/1,235)	100% (1,235/1,235)	100% (874/874)
ESBL mechanism of resistance	NA (0/0)	NA (0/0)	Not targeted	100% (3/3)	100% (3/3)	Not targeted
Carbapenemase mechanisms of resistance (five targets)	NA (0/0)	NA (0/0)	Not targeted	100% (15/15)	100% (15/15)	Not targeted
Cumulative organism target detection	100% (36/36)	100% (25/25)	100% (27/27)	99.7% (1,974/1,979)	99.2% (1,974/1,990)	99.7% (1,303/1,307)
Off-panel targets						
*Staphylococcus epidermidis*	0% (0/4)	0% (0/4)	0% (0/1)	100% (61/61)	100% (61/61)	100% (45/45)
*Staphylococcus capitis*	0% (0/1)	0% (0/1)	NA (0/0)	100% (64/64)	100% (64/64)	100% (46/46)
*Bacillus* species	0% (0/1)	0% (0/1)	NA (0/0)	100% (64/64)	100% (64/64)	100% (46/46)
Total samples evaluated	65	65	46	65	65	46

^*a*^ SOCj, standard-of-care joint fluid microbiologic studies (including joint fluid culture with or without 16S PCR/S, as ordered by the clinical team); MRSA, methicillin-resistant *S. aureus*; ESBL, extended-spectrum beta-lactamase; 16S PCR/S, 16S rRNA PCR followed by Sanger sequencing.

^*b*^ NA, no percentage is calculated when the denominator is 0.

The cumulative negative percent agreement for on-panel organism targets by the BIOFIRE JI panel was 99.7% (1,974/1,979), 99.2% (1,974/1,990), and 99.7% (1,303/1,307) compared to detection by SOCj, joint fluid culture only, or 16S PCR/S only, respectively (Table 3). The BIOFIRE JI panel detected four organisms that were not detected by SOCj, including two samples with *K. kingae*, one sample with *S. pneumoniae*, and one sample with *Salmonella* species. These four organisms corresponded to five detected targets as both the genus-level *Streptococcus* target and the species-level *S. pneumoniae* target were detected in one sample.

### Sample-level analytic performance evaluation of the BIOFIRE JI panel

When comparing the sample-level agreement for on-panel organisms, the BIOFIRE JI panel yielded 100% positive agreement to the SOCj results (29/29) and 100% positive agreement compared to 16S PCR/S alone (21/21). However, the sample-level negative agreement for on-panel organisms was 89% (32/36) compared to SOCj and 80% (20/25) compared to 16S PCR/S alone. The lower negative percent agreement was due to additional organism detections by the BIOFIRE JI panel and the detection of one yeast that is not targeted by 16S PCR/S but was detected in culture. Interestingly, positive organism detection was higher by both SOCj (OR 4.2, 95% CI 1.4–11.7; *P* = 0.01) and the BIOFIRE JI panel (OR 3.8, 95% CI 1.2–10.5; *P* = 0.02) in patients who received antibiotics within 24 h before the joint fluid was collected compared to those who did not receive antibiotics in the preceding 24 h.

The sample-level detection of on-panel organisms by the BIOFIRE JI panel compared to cSOC (including non-joint fluid microbiology tests such as bone or other joint tissue cultures) yielded 91% (30/33) positive agreement and 91% (29/32) negative agreement. In three samples, an on-panel organism was detected in bone culture but not in joint fluid studies [*S. pyogenes*, methicillin-susceptible *S. aureus* (MSSA), and *K. kingae*], and three organisms were detected by the BIOFIRE JI panel but not by cSOC (two *K. kingae* and one *S. pneumoniae*).

The most common organism detected by all methods was *S. aureus*. When recovered in the joint fluid culture, methicillin resistance was evaluated by phenotypic methods (PBP2a production and oxacillin and cefoxitin microbroth dilution). The BIOFIRE JI panel includes the gene targets that confer resistance to methicillin, *mecA/C* and MREJ. Of the four samples from which methicillin-resistant *S. aureus* (MRSA) was detected by phenotypic methods in culture, *mecA/C* and MREJ were detected by the BIOFIRE JI panel in three (75% positive percent agreement for predicting MRSA, 3/4; Table 3). ESBL-producing or carbapenemase-producing gram-negative organisms were not recovered in culture or predicted by the BIOFIRE JI panel from any of the tested samples. Additionally, no samples were found to contain *Enterococcus* species during this study. Therefore, the *vanA/B* target performance was not evaluated.

### Predicted potential clinical impact

The potential clinical impact of the BIOFIRE JI panel results was assessed by three adjudicators using a predefined, study-specific rubric (see *Methods* and Table 1). Had the BIOFIRE JI panel results been available for clinical use within 3 h of sample collection, we predicted that 27.7% (18/65) of test results could have had a positive impact, 1.5% (1/65) a negative impact, 66.2% (43/65) no impact, and 4.6% (3/65) unknown impact. None of the cases with a negative BIOFIRE JI panel result were adjudicated to have had an impact. This was largely driven by the study-specific adjudication criterium that assumed that other clinical or laboratory results (e.g., joint fluid culture) would be required before making a clinical diagnosis of infectious versus non-infectious cases. Among the cases with potential positive impact, one was high positive, nine moderately positive, and eight low positive (Table 4). Thirteen cases were adjudicated to have had a potential positive impact due to the opportunity to optimize therapy from an adequate empiric regimen. In five cases, the patient was not on adequate therapy, and the BIOFIRE JI panel results could have informed the selection of a more appropriate treatment sooner. One case was adjudicated as high positive impact because empirical therapy was suboptimal, and we predicted that obtaining a faster organism identification would have enabled discharge from the hospital 1 day sooner in an otherwise stable patient completing a course of intravenous antibiotics for *Neisseria gonorrhoeae* joint infection. One case was adjudicated to potentially have had a negative impact due to the absence of detecting *mecA/C* and MREJ in a sample that yielded MRSA in culture.

**TABLE 4:** Potential clinical impact case adjudication summary^*a*^

BIOFIRE JI Panel organism detection	Total samples	Potential clinical impact
*Staphylococcus aureus*	14	Moderately positive (1/14), low positive (5/14), negative (1/14), and no impact (7/14)
*Kingella kingae*	5	Moderately positive (3/5); unknown (2/5)
*Streptococcus pyogenes*	4	Low positive (3/4); no impact (1/4)
*Streptococcus pneumoniae*	2	Moderately positive (1/2); unknown (1/2)
*Streptococcus agalactiae*	1	Moderately positive (1/1)
*Salmonella* species	2	Moderately positive (1/2); no impact (1/2)
*Escherichia coli*	2	No impact (2/2)
*Staphylococcus lugdunensis*	1	Moderately positive (1/1)
*Neisseria gonorrhoeae*	1	High positive (1/1)
*Candida albicans*	1	Moderately positive (1/1)
No organism detected	32	No impact (32/32)

^*a*^ JI, joint infection.

## DISCUSSION

The BIOFIRE JI panel has been limitedly evaluated in pediatric patients. Udaondo and colleagues evaluated 50 joint fluid samples from pediatric patients in Spain, of which 74% were from patients under 3 years of age (15). Only *K. kingae, S. aureus*, and *S. pyogenes* targets were detected by the BIOFIRE JI panel during the study. This study showed that patients 6 months to 3 years old who were in good general condition and lacked specific risk factors could safely and effectively be treated with oral antibiotics without surgical intervention based, in part, on the rapid panel results. Camacho-Moreno and colleagues evaluated 32 patients with a 75% positivity rate, including six detections by the BIOFIRE JI panel only (2 *S. aureus*, 2 *S. pyogenes*, 1 *K. kingae,* and 1 *C. albicans*) (16). *S. aureus* and *S. pyogenes* were the most common pathogens detected in this study with a high proportion of MRSA (11/19). Our study remains relatively small (65 samples) but spanned the complete range of pediatric ages and evaluated twelve organism targets and one resistance determinate, which is the broadest evaluation in an exclusively pediatric population, to our knowledge. Nineteen organisms and six resistance mechanisms targeted by the panel were not observed in our study or other published pediatric studies, indicating that these organisms/mechanisms are extremely uncommon in this age group. In contrast to adults, pediatric joint infections are primarily of the native joint. *K. kingae* and *Streptococcus* species are common causes of infection, whereas *Staphylococcus epidermidis* is an infrequent cause of infection. A consistent finding among pediatric and adult patients and native or prosthetic joints is that *S. aureus* is a predominant cause of joint infections (10, 13, 16, 17).

In this study, there were several discrepancies between the BIOFIRE JI panel and SOCj (Table S1). In three cases, the detection of additional organisms by the BIOFIRE JI panel was plausible, but no cSOC microbiologic results supported their detection. In the fourth case, *Salmonella* species was detected from a patient already being treated for multifocal *Salmonella* joint infection. Six samples grew an organism in culture that was not targeted by the BIOFIRE JI panel. *S. capitis*, *Bacillus* species, and one case of *S. epidermidis* were interpreted to be likely contaminants. Three cases of *S. epidermidis* were interpreted to be clinically significant by the treating team. The pediatric orthopedic physicians at our institution typically consider whether *S. epidermidis* was detected by culture from multiple specimens, rather than a single joint fluid, the quantity of *S. epidermidis* recovered in culture (e.g., a single colony on one plate only versus multiple colonies), and the patient’s risk factors when determining the clinical significance of this organism in musculoskeletal specimens. Therefore, the absence of *S. epidermidis* from the BIOFIRE JI panel was not considered a major test limitation but does highlight the importance of performing culture in addition to a molecular method such as the BIOFIRE JI panel.

Another interesting finding in this study was that samples from patients who received antibiotics within the 24 h preceding joint fluid collection were significantly more likely to be positive. We speculate that this may be due to a higher severity of illness in positive patients, which also drove the clinical decision to start antibiotic therapy before the joint fluid collection. A limitation of this study is that antibiotics were only evaluated in the preceding 24 hours. If the efficacy of the antibiotics for a longer time preceding the joint fluid collection was evaluated, the relative yield of culture, 16S PCR/S, and/or BIOFIRE JI panel may show differences after exposure to antibiotics.

For the potential clinical impact adjudication evaluations, we assumed the BIOFIRE JI panel results would be available within 3 h of sample collection. During the study, the average hands-on sample processing time was 7 minutes and the on-instrument run time was 56 minutes per sample. The additional 2 h built into the adjudication criteria accommodates flexibility in a laboratory’s workflow for setting up samples and reporting results. Based on the application of our study rubric (Table 1), all the adjudicated positive clinical impact cases were due to the estimated faster turnaround time of the BIOFIRE JI panel results, enabling targeted antimicrobial therapy sooner. In comparison to the estimated 3 h turnaround time for the BIOFIRE JI panel, the average turnaround time for 16S PCR/S results during this study was 8.9 d [range 4.9–19.8 d] and the average time to identification of all detected organisms in positive cultures was 1.4 d [range 0.6–3.2 d]. Laboratories may consider selecting different algorithms for using the BIOFIRE JI panel according to the optimal balance of resource utilization and clinical impacts determined at their institution.

The detection of certain organisms was disproportionately associated with being predicted to have a positive potential clinical impact. Specifically, in all three cases where *K. kingae* was detected by SOCj, the faster positive detection by the BIOFIRE JI panel was adjudicated to carry a moderately positive potential impact. In three of the four cases of *S. pyogenes*, low positive potential impact was predicted. On the other hand, *S. aureus* was the most common organism detected, but only 6/14 cases had positive potential impact (one moderately positive and five low positive) and one potentially negative impact. Interestingly, organisms that were only detected once or twice each during this study were commonly adjudicated to have a positive potential impact (*S. agalactiae, S. pneumoniae, S. lugdunensis*, *Salmonella* species, and *C. albicans*). This may suggest that empiric therapy regimens at our institution are suboptimal for many of the infrequent causes of joint infection, leaving opportunity for positive clinical impact if detected more quickly.

In one case, an isolate of *S. aureus* was phenotypically resistant to cefoxitin when tested by broth microdilution, but *mecA*/*C* and MREJ were not detected on the BIOFIRE JI panel. The isolate recovered in culture was negative for PBP2a antigen detection, and the *mecA* gene was not detected when tested by a second molecular test. Its resistance to beta-lactam agents was likely due to a non-*mecA* mechanism. This is a known limitation to using the absence of *mecA* detection, alone, to predict MSSA. The sensitivity of predicting MRSA by direct detection of *mecA* gene determinants in positive blood cultures has been reported to be 93.8%–98.6% (18–20). In this study, 10/11 (91%) *mecA/C* and MREJ-negative samples correlated with isolates that were phenotypically MSSA. While using the absence of *mecA* to predict MSSA carries the risk of overinterpreting the presence of MSSA, this rapid result also enables optimization of treatment for MSSA more quickly and contributed to five cases having low positive and one case with moderate positive potential impact in this study.

This study had a few limitations. First, bias may have been introduced due to the enrollment strategy: i) identification of candidates by the orthopedic provider depended, in part, on the timing of first seeing the patient versus the procedure and having enough time to coordinate enrollment, and ii) retrospective sample selection was based on the availability of residual joint fluid, thus skewing toward higher volume collections. Second, the potential clinical impact was determined through clinical chart review and adjudication of what may have happened had the BIOFIRE JI panel been used for clinical care. Predefined adjudication criteria were applied to improve study transparency and consistency between adjudicators. However, since the BIOFIRE JI panel results were not provided for clinical use, independent interpretations and assumptions were required by the adjudicating study personnel.

### Conclusions

The BIOFIRE JI panel had a high positive agreement with culture and 16S PCR/S and detected the same organism in all culture or 16S PCR/S-positive samples when targeted by the panel. The BIOFIRE JI panel detected all organisms detected by 16S PCR/S, but not by culture during the study period. Therefore, this panel may be considered a reasonable alternative to 16S PCR/S and enables more rapid organism detection. Finally, the rapid time to results by the BIOFIRE JI panel has the potential to support the optimization of antimicrobial therapy sooner.

## SUPPLEMENTAL MATERIAL

Table S1 jcm.00278-25-s0001.docx Overview of Discrepant Results between the BIOFIRE JI panel and SOCj.

ASM does not own the copyrights to Supplemental Material that may be linked to, or accessed through, an article. The authors have granted ASM a non-exclusive, world-wide license to publish the Supplemental Material files. Please contact the corresponding author directly for reuse.
