# Supplementary material for: Performance evaluation of a commercial multiplex pathogen panel for the diagnosis of pediatric joint infections
Source: J Clin Microbiol. 2025 Jun 2;63(7):e00278-25. doi: 10.1128/jcm.00278-25 (PMC12239719; doi:10.1128/jcm.00278-25)
Supplement: Table S1 — Overview of Discrepant Results between the BIOFIRE JI panel and SOCj. [file jcm.00278-25-s0001.docx]

**Supplemental Table 1: Overview of Discrepant Results between the BIOFIRE**® **JI panel and SOCj**

| **SOCj Results** | **BIOFIRE**® **JI Panel Result** | **Type of Discrepancy** | **Potential Clinical Impact** | **Comments** |
| --- | --- | --- | --- | --- |
| Culture: No organism detected 16S PCR/S: No organism detected | *Kingella kingae* detected | Additional detection by BIOFIRE® JI panel | Unknown Impact | Orthopedic clinical notes indicated that empiric treatment was selected to provide *K. kingae* coverage based on age, clinical findings, and negative cSOC test results. Ceftriaxone and clindamycin inpatient treatment (x3 days) followed by trimethoprim-sulfamethoxazole outpatient (x3 weeks) with clinical resolution of septic arthritis following treatment. |
| Culture: No organism detected 16S PCR/S: No organism detected | *Kingella kingae* detected | Additional detection by BIOFIRE® JI panel | Unknown Impact | Orthopedic clinical notes indicated that empiric treatment was selected to provide *K. kingae* coverage based on age, clinical findings, and negative cSOC test results. Ceftriaxone and clindamycin only given immediately after the procedure, then switched to trimethoprim-sulfamethoxazole outpatient (x4 weeks) with clinical resolution of septic arthritis and osteomyelitis following treatment. |
| Culture: No organism detected 16S PCR/S: Not performed | *Salmonella* species detected | Additional detection by BIOFIRE® JI panel | No Impact | The patient was diagnosed with multifocal *Salmonella* infection 2.5 weeks before collection of the enrolled sample and was receiving targeted antimicrobial therapy. Culture and 16S PCR/S were positive from joint fluid obtained from the same joint during the initial procedure, before antibiotic treatment. Joint fluid specimens collected from other joints during the same procedure were positive for *Salmonella* by culture. |
| Culture: No organism detected 16S PCR/S: Not performed | *Streptococcus* sp; *Streptococcus pneumoniae* detected | Additional detection by BIOFIRE® JI panel | Unknown Impact | Orthopedic clinical notes indicated that empiric treatment was selected to provide *Kingella kingae* coverage based on age, clinical findings, and negative cSOC test results. Ceftriaxone and clindamycin inpatient treatment followed by trimethoprim-sulfamethoxazole outpatient (x3 weeks) with clinical resolution following treatment. *Streptococcus pneumoniae* was not specifically targeted for treatment in this patient. |
| Culture: methicillin resistant *Staphylococcus aureus* detected 16S PCR/S: *S. aureus* detected | *Staphylococcus aureus* detected; *mecA/C* and MREJ not detected | Resistance determinant target(s) on BIOFIRE® JI panel did not predict phenotypic resistance | Negative Impact | *S. aureus* was isolated in culture and initially reported as MSSA based on a negative PBP2a antigen result. The isolate was reproducibly, phenotypically resistant to cefoxitin by broth microdilution. The culture report was corrected to MRSA. The clinical team targeted therapy to MSSA when reported and modified therapy upon the corrected report. A BIOFIRE® JI panel detection of "*S. aureus*, *mecA/C* and mreJ not detected" may have led to suboptimal antibiotic therapy ~16 h earlier. |
| Culture: *Staphylococcus epidermidis* detected 16S PCR/S: No organism detected | No organism(s) detected | Off-panel organism detected by culture | No Impact | *S. epidermidis* was interpreted to be a contaminant by the treating team. *S. epidermidis* was isolated from inoculated broth medium only and was resistant to clindamycin. The patient was treated empirically with trimethoprim-sulfamethoxazole for 2 weeks based on age and clinical findings with inadequate clinical response. Treatment was empirically changed to clindamycin x 3 additional weeks with clinical resolution. |
| Culture: *Staphylococcus epidermidis* detected 16S PCR/S: Not performed | No organism(s) detected | Off-panel organism detected by culture | No Impact | *S. epidermidis* was interpreted to be the cause of infection by the treating team and was treated. Patient with history of osteosarcoma with radical resection, placement of protheses, and a joint allograft. *S. epidermidis* was also isolated from tissue and abscess specimen cultures collected from the same procedure. |
| Culture: *Staphylococcus epidermidis* detected 16S PCR/S: *Staphylococcus epidermidis* detected | No organism(s) detected | Off-panel organism detected by culture and 16S PCR/S | No Impact | *S. epidermidis* was interpreted to be the cause of infection by the treating team and was treated with clinical resolution. Patient status post recent ACL reconstruction surgery. *S. epidermidis* was also recovered from tissue and a second joint fluid specimen from the same joint, on the same day. |
| Culture: methicillin susceptible *Staphylococcus aureus* and *Staphylococcus epidermidis* detected 16S PCR/S: *Staphylococcus aureus* detected | *Staphylococcus aureus* detected; *mecA/C* and MREJ not detected | Off-panel organism detected by culture | No Impact | MSSA and *S. epidermidis* were both interpreted to be the causes of infection by the treating team and were treated with clinical resolution. The patient was diagnosed with septic arthritis after a contaminated wound infection due to trauma with a machete. Both organisms were isolated by culture from the enrolled joint fluid and in a specimen collected from the inciting wound. |
| Culture: methicillin susceptible *Staphylococcus aureus* and *Staphylococcus capitis* detected 16S PCR/S: No organisms detected | *Staphylococcus aureus* detected; *mecA/C* and MREJ not detected | Off-panel organism detected by culture | No Impact | MSSA, alone, was interpreted to be the cause of infection by the treating team (including infectious disease consultation) and was treated with clinical resolution. The patient was a 15-week-old infant with no significant past medical history. |
| Culture: *Bacillus* species detected 16S PCR/S: *Kingella kingae* detected | *Kingella kingae* detected | Off-panel organism detected by culture | Moderate positive | *K. kingae*, alone, was interpreted to be the cause of infection by the treating team and was treated with clinical resolution. The patient was 2-year-old with no significant past medical history. |

JI, joint infection; SOCj, SOCj, standard of care joint fluid microbiologic studies (including joint fluid culture with or without 16S PCR/S, as ordered by the clinical team); 16S PCR/S, 16S rRNA PCR followed by Sanger sequencing; cSOC, complete standard of care microbiology testing (included all microbiologic testing that contributed to treatment decisions);
